# Greater Intake of Fruit and Vegetables Is Associated with Greater Bone Mineral Density and Lower Osteoporosis Risk in Middle-Aged and Elderly Adults

**DOI:** 10.1371/journal.pone.0168906

**Published:** 2017-01-03

**Authors:** Rui Qiu, Wen-ting Cao, Hui-yuan Tian, Juan He, Geng-dong Chen, Yu-ming Chen

**Affiliations:** Department of Medical Statistics and Epidemiology, Guangdong Provincial Key Laboratory of Food, Nutrition and Health, School of Public Health, Sun Yat-sen University, Guangzhou, People's Republic of China; Leibniz-Institut fur Pflanzengenetik und Kulturpflanzenforschung Gatersleben, GERMANY

## Abstract

**Objective:**

A few studies have suggested that the consumption of fruit and vegetables (FV) may benefit bone health, but limited data are available in Asian subjects. We examined the association between FV intake and bone mineral density (BMD) and osteoporosis in Chinese adults.

**Methods:**

This population-based cross-sectional study involved 2083 women and 1006 men aged 40–75 years in Guangzhou, China. Habitual dietary data was collected from a 79-item food frequency questionnaire by face-to-face interviews. The BMD was measured for the whole body (WB), lumbar spine (LS), total hip (TH) and femur neck (FN) with dual-energy X-ray absorptiometry.

**Results:**

After adjustment for potential covariates, we observed dose-dependent associations between total FV intake and BMD and osteoporosis risk. The mean BMD was higher in tertile 3 *vs*. tertile 1 by 1.33% (TH) and 1.31% (FN) for FV, and 1.10% (WB), 1.57% (TH), and 2.05% (FN) for fruit (all *P*-trends < 0.05). Significant beneficial associations with BMD at some sites were also found in most fruit categories but not in total vegetables or their subgroups. The odds ratios (95% confidence interval) of osteoporosis (T-score ≤ −2.5) in tertile 3 (*vs*. 1) were 0.73 (0.58–0.92), 0.37 (0.22–0.60), and 0.71 (0.52–0.97) for FV; 0.82 (0.66–1.03), 0.48 (0.30–0.77) and 0.89 (0.61–1.12) for fruit; and 0.80 (0.64–1.01), 0.57 (0.35–0.92) and 0.76 (0.55–1.05) for vegetables at the LS, TH, and FN, respectively. The favorable association between FV intake and the occurrence of osteoporosis was evident only in subjects with lower BMI (<24.0 kg/m^2^, *P-trends* < 0.05).

**Conclusions:**

Greater intake of FV was independently associated with a higher BMD and a lower presence of osteoporosis in middle-aged and elderly Chinese subjects with lower BMI. Fruit tended to have more contribution to the favorable association than vegetables.

## Introduction

Osteoporosis is an emerging medical and socioeconomic threat worldwide [1]. Nutrition is one of several important modifiable factors for optimal bone health and prevention of osteoporosis [2]. Despite that previous studies mainly focused on the roles of calcium, vitamin D, protein, and dairy and soya products, increasing evidence suggests a positive association between fruit and vegetable (FV) components and bone health [2–4]. These components include potassium, manganese, vitamin B complex, vitamins C, E, and K, and phytochemicals (e.g., carotenoids) [4].

Several epidemiological studies have investigated the association between FV intake and bone health. Most observational studies have found that greater intake of FV is associated with an increase in bone mass and decreases in bone loss and fracture risk [5–11]. An intake below the recommended five servings/day of FV confers higher rates of hip fracture in a Swedish cohort study of 40,644 men and 34,947 women after 14 years follow-up [5]. Similar favorable association between FV intake and hip fracture was also found in a prospective European cohort of 48,814 men and 139,981 women [9]. However, conflicting results were found in other studies [12–14] A randomized controlled trial of 276 healthy postmenopausal women showed that supplementation with 300 g of FV per day for 2 years did not significantly and persistently reduce bone turnover or increase bone mineral density (BMD) [13]. In addition, most of these studies were performed in Western countries and mainly focused on postmenopausal women.

The typical Chinese diet is plant-based and thus differs from those of many Western populations. A Chinese national survey showed a higher intake of FV (320 g/d) in Chinese subjects than in American subjects (about 256 g/d for men and 280g/d for women) [15]. The prevalence of osteoporosis among Chinese subjects remains low compared to that in Caucasian populations, but less evidence on the role of FV in bone health is developed from Chinese populations [6, 7]; therefore, it remains uncertain whether a higher intake of FV is associated with better bone health in Chinese subjects. Although the prevalence of osteoporosis is approximately 50% lower in men than women, a higher mortality rate was observed in men after osteoporotic fracture [16]. However, research in men was less sufficient. Determination of the role of FV intake in bone health in aging men and women is clearly an important objective that has implications for the establishment of nutritional guidelines for the management of osteoporosis. This study was performed to examine the association between FV intake and BMD and osteoporosis risk in middle-aged and elderly Chinese men and women.

## Materials and Methods

### Study participants

This cross-sectional study was based on the Guangzhou Nutrition and Health Study (GNHS), a community-based prospective cohort study designed to investigate the nutritional determinants of cardiometabolic outcomes and osteoporosis. The participants’ criteria and the recruitment procedure for this study have been described in detail elsewhere [17]. Briefly, 3169 apparently healthy participants aged 40 to 75 years were enrolled at baseline and completed a detailed questionnaire survey (diet and covariates) between 2008 and 2010 in Guangzhou, China. A total of 2520 (79.5%) participated in the first follow-up and completed the same questionnaire and additional BMD measurements between 2011 and 2013. In 2013, 879 new participants were recruited in the same manner, and completed the same questionnaire and BMD measurements. We excluded those with the following conditions: (1) a confirmed history of parathyroid and thyroid disorder, diabetes, fractures, malignancy, cardiovascular disease, and other chronic diseases (257 participants), (2) missing dietary data or BMD data (40 participants), and (3) without a reasonable range of energy intake(500–4000 kcal/d for men, 500–3500 kcal/d for women [18, 19]) (13 participants). Finally, 3089 participants who completed at least one dietary survey and the BMD tests were included in analyzing the cross-sectional association between dietary FV intake and BMD/osteoporosis (S1 Fig). Average dietary data were used for the analysis for those with twice dietary assessments. The study was approved by Sun Yat-Sen University’s School of Public Health Ethics Committee, and was conducted according to the Declaration of Helsinki. Written informed consent was obtained from all participants.

### General data collection

Face-to-face interviews conducted by trained interviewers based on a structured questionnaire were used to collect data on sociodemographic characteristics, lifestyle factors, habitual dietary intake, calcium and multivitamin supplement use, years since menopause (for women), medical history, medications, and physical activity (including activities at and after work, exercise, and others) [20]. The metabolic equivalent for task (MET) hours per day (excluding sleeping and sitting) was calculated on the basis of a 19-item questionnaire for physical activity [21].

#### Dietary assessment

A validated 79-item quantitative food frequency questionnaire (FFQ) was used to assess habitual dietary intake [22]. For each food item, five possible frequencies (never, per year, per month, per week, and per day) and one quantitative response (average amounts) were available. For seasonal foods, participants were asked to report how many months of the year they consumed each item. The reference period was the year before the interview. Daily intakes were calculated according to the 2002 China Food Composition [23]. Food photographs were provided as visual aids to help participants recall portion sizes. The validity was assessed by using correlation coefficients between the FFQ and six 3-day dietary records (0.37 for vegetables and 0.56 for fruit) [22]. For the followed up subjects, average values of dietary intake at both baseline and follow-up were used to better estimate their long-term intake. For the new participants, only once dietary data was collected and available for further analyses.

The FV groups or items listed on the FFQ have been described previously [24]. More specifically, the fruit groups or items included 1) citrus fruit; 2) apples, pears, peaches, pineapples, and plums; 3) bananas; 4) grapes; 5) lychee and longan; 6) mangoes and persimmons; 7) papaya; 8) cantaloupes, watermelons, and other muskmelons; 9) durian; and 10) other fruit. The vegetable groups or items included 1) fresh beans; 2) dark green leafy vegetables; 3) light green leafy vegetables; 4) onion and garlic; 5) turnips, eggplant, and melon vegetables; 6) tomatoes; 7) peppers; 8) carrots; 9) starchy tubers (e.g., yams, potatoes, lotus root, etc.,); 10) fresh corn; 11) pickles; and 12) mushrooms and fungi. Starchy and pickled vegetables were excluded from the vegetable intake calculation. We classified FV into six vegetable subgroups and six fruit subgroups (S1 Table) on the basis of the similarity of their nutrient composition.

#### Anthropometric and BMD measurements

The participants’ height and weight were measured to the nearest 0.1 cm and 0.1 kg, respectively, in light clothing and no shoes. The body mass index (BMI, in kg/m^2^) was calculated. Dual-energy X-ray absorptiometry (Discovery W; Hologic Inc., Waltham, MA, USA) was used to measure the BMD (g/cm^2^) of the whole body (WB), the lumbar spine (LS, L1-L4), the total hip (TH), and the femur neck (FN) [17]. The in vivo coefficients of variation of the measurements in 30 participants after repositioning were 0.87%, 1.02%, 1.92%, and 1.18%, at the four bone sites, respectively. The long-term coefficient of variation was 0.26% as calculated by testing the phantom daily between March 2011 and May 2015. Osteoporosis is defined as a T-score of less than −2.5 according to the reference values of 30-year-old white women from the dual-energy X-ray absorptiometer manufacturer’s reference database for the LS [25] and those of 20- to 29-year-old non-Hispanic white women from NHANES III for the hip [26].

### Statistical analysis

All data were entered twice using Epidata 3.1. The missing values (n, %) were replaced by the means or mode of the participants for the following variables: BMI (1, 0.03%), education level (12, 0.39%), marital status (1, 0.03%), income (7, 0.23%); years since menopause (set at 0 for men, 14, 0.45% for women), estrogen use (set as no for men, 15, 0.49% for women), antiosteoporosis drug use (15, 0.49%); calcium supplement use (2, 0.06%), multivitamin supplement use (3, 0.10%), passive smoking (3, 0.10%), tea consumption (2, 0.06%), and alcohol consumption (1, 0.03%). Box-Cox [27], logarithmic or square root transformation was used for dietary intake to achieve an approximately normal distribution as appropriate. The dietary intake (g/day) of fruit, vegetables, calcium (excluding calcium from FV), and protein were adjusted for total energy intake using the residual method after the transformation [28]. We replaced BMD outliers (number: 12 [WB], 15 [LS], 3 [TH], and 17 [FN]) that were at least 3 SD away from the sex-specific mean with the next-most-extreme value.

The subjects were classified into sex-specific tertiles by energy-adjusted FV intake and their total values. One-way analysis of variance (ANOVA) and analyses of covariance (ANCOVA) were used to compare the mean BMD difference among the tertiles. In ANCOVA we adjusted for age; sex; BMI; education level; marital status; income; years since menopause (set at 0 for men); estrogen use (set as no for men); antiosteoporosis drug use; dietary intake of energy, protein, and calcium; calcium and multivitamin supplement use; smoking; passive smoking; tea and alcohol consumption; and physical activity (in MET, hour/day). Pairwise comparisons were made with the Bonferroni method. Linear trend was also tested for the intake tertiles and BMD. To examine the possible different associations between specific FV categories and BMD, ANCOVA was also conducted for different FV subgroups.

Logistic regression analyses were used to test the independent association of FV levels with the occurrence of osteoporosis after controlling for the covariates as mentioned in ANCOVA, and the bottom tertile (T1) was defined as the reference group. To test for linear trends, the ordinal values for the categories of dietary intake were treated as continuous variables. We conducted subgroup analyses in which subjects were stratified by sex, age, education, income, BMI, MET, smoking, tea or alcohol consumption, use of calcium or multivitamin supplements, and osteoporosis treatment, respectively, and the interaction terms were examined simultaneously.

A two-sided *P* value of less than 0.05 was considered to be statistically significant. SPSS for Windows, version 19.0 by IBM (SPSS Inc., Chicago, Illinois, USA) was used for the analyses.

## Results

### Participant characteristics

Table 1 shows the characteristics of the participants. The 2083 women and 1009 men had mean ages of 59.7 and 62.3 years, respectively. The men had higher BMIs than the women and ate less FV. The median intake of total fruit, total vegetables, and FV combined were 141, 343, and 499 g/d for women and 109, 321, and 434 g/d for men, respectively. Approximately half of the fruit intake was from the group including apples, pears, peaches, pineapples, and plums, followed by citrus fruit and bananas (S1 Table). Of the total vegetable intake, about 44% was dark-green vegetables, and 17% consisted of carrots, tomatoes, and peppers.

**Table 1:** Characteristics of study participants.

	Women(n = 2083)	Men(n = 1006)	*P*
Age (years)	59.7±5.4	62.3±6.6	<0.001
Height (cm)	155.1±5.4	166.1±5.9	<0.001
Weight (kg)	56.3±8.2	66.2±9.8	<0.001
Married participants, n (%)	1766(84.8)	972(96.6)	<0.001
BMI (kg/m^2^)	23.4±3.2	23.9±3.0	<0.001
Household income (yuan/month/person), n (%)			
≤2000	1031(49.5)	389(38.7)	
2001–3000	518(24.9)	280(27.8)	
≥3001	534(25.6)	337(33.5)	<0.001
Education level, n (%)			
Secondary school or below	606(29.1)	276(27.4)	
High school	1036(49.7)	389(38.7)	
College or above	441(21.2)	341(33.9)	<0.001
Years since menopause (years)	9.8±6.1		
Estrogens user, n (%)	131(6.3)		
Osteoporosis treatment user, n (%)	114(5.5)	7(0.7)	
Smoker ^a^, n (%)	8(0.4)	185(18.4)	<0.001
Passive smoker ^b^, n (%)	551(26.5)	200(19.9)	<0.001
Alcohol drinker ^c^, n (%)	77(3.7)	175(17.4)	<0.001
Tea drinker ^d^, n (%)	1005(48.2)	749(74.5)	<0.001
Calcium supplement use ^e^, n (%)	710(34.1)	208(20.7)	<0.001
Multivitamin supplement use ^e^, n (%)	444(21.3)	131(13.0)	<0.001
Daily dietary intake			
Energy (kcal)	1595(461)	1834(597)	<0.001
Diet calcium (mg)	663(264)	642(307)	0.025
Diet protein (g)	66.8(23.0)	72.8(26.6)	<0.001
Vegetables (g)	343(194)	321(184)	0.001
Fruit (g)	141(119)	109(100)	<0.001
Fruit and vegetables (g)	499(261)	434(251)	<0.001
Physical activity (MET·h/d)	17.5±6.6	15.7±7.2	<0.001

The data were presented as means ± standard deviation (SD), or medians and interquartile range (IQR) for the continuous variables, as appropriate. Categorical variables were presented as number (%).

BMI, body mass index; MET, metabolic equivalent for task.

^a^ Smokers are defined as having smoked ≥1 cigarette daily for at least 6 consecutive months.

^b^ Passive smokers are defined as exposure to other peoples’ tobacco smoke for at least 5 min daily in previous 1 year.

^c^ Alcohol drinkers are defined as having had wine ≥1 time(s) daily for at least 6 consecutive months.

^d^ Tea drinkers are defined as drinking ≥2 cup of tea per week.

^e^ Calcium or multivitamin supplement users are defined as having used the supplements ≥30 times.

### Covariate-adjusted BMD by the FV tertiles

After adjustment for potential confounding factors, ANCOVA in Table 2 shows that statistically significant, dose-dependent positive relationships with BMD were found for total FV intake and fruit intake. The mean BMD was higher in tertile 3 *vs*. tertile 1 by 1.33% (or 0.10 SD) and 1.31% (or 0.09 SD) for FV at the TH and FN, and 1.10% (or 0.11 SD), 1.57% (or 0.12 SD), and 2.05% (or 0.13 SD) for fruit at the WB, TH, and FN (all *P*-trends < 0.05), respectively. The favorable associations tended to be more pronounced in the unadjusted model by using ANOVA (S2 Table). The results were largely similar to those of univariable analysis (ANOVA) after adjustment for the all other covariates as mentioned above except BMI (S3 Table), but substantially attenuated after further adjusted for BMI (Table 2). No significant association was observed between the intake of total vegetables or their subgroups and BMD at any of these sites (Tables 2 and 3). All fruit subgroups except persimmons, mangoes, durian and melon fruit showed significant positive associations with BMD at some sites (*P* trend: 0.001 to 0.047) (Table 3).

**Table 2:** Adjusted mean of BMD at various sites by fruit and vegetables intake tertiles of the study participant ^a^.

BMD	T1 (n = 1029)		T2 (n = 1031)		T3 (n = 1029)		Difference ^*b*^		ANCOVA	
g/cm^2^	Mean	SE	Mean	SE	Mean	SE	Abs.	%	*P* difference	*P* trend
**Total fruit and vegetable intake**										
Whole body	1.095	0.003	1.102	0.003	1.103	0.003	0.008	0.73	0.147	0.072
Spine (L1–L4)	0.880	0.004	0.887	0.004	0.889	0.004	0.009	1.02	0.265	0.131
Total hip	0.825	0.003	0.833	0.003	0.836	0.003*	0.011	1.33	0.051	**0.016**
Femoral neck	0.685	0.003	0.690	0.003	0.694	0.003	0.009	1.31	0.112	**0.036**
**Fruit intake**										
Whole body	1.095	0.003	1.097	0.003	1.107	0.003*	0.012	1.10	0.016	**0.008**
Spine (L1–L4)	0.880	0.004	0.887	0.004	0.889	0.004	0.010	1.14	0.240	0.110
Total hip	0.826	0.003	0.830	0.003	0.839	0.003**	0.013	1.57	0.010	**0.003**
Femoral neck	0.683	0.003	0.689	0.003	0.697	0.003**	0.014	2.05	0.006	**0.001**
**Vegetable intake**										
Whole body	1.101	0.003	1.098	0.003	1.100	0.003	-0.001	-0.09	0.692	0.744
Spine (L1–L4)	0.884	0.004	0.884	0.004	0.888	0.004	0.004	0.45	0.744	0.553
Total hip	0.830	0.003	0.828	0.003	0.836	0.003	0.006	0.72	0.211	0.193
Femoral neck	0.688	0.003	0.688	0.003	0.693	0.003	0.005	0.73	0.426	0.282

BMD, bone mineral density. SE, standard error. ANCOVA, analyses of covariance.

* *P*<0.05

** *P*<0.01

***P<0.001, Compared with tertile 1.

^*a*^ Covariates adjusted for in the multivariate model: age, sex, BMI, educational level, marital status, household income, years since menopause (set at 0 for men), estrogen use (set as no for men), osteoporosis treatment use, physical activities (MET), smoking, passive smoking, tea and alcohol drinking, use of Calcium supplement, use of multivitamin supplement, dietary energy, energy-adjusted diet protein and diet Calcium (remove the calcium from FV group being analyzed).

^*b*^. Difference between tertile 3 and tertile 1: Abs., absolute mean difference (T3–T1); %, relative difference, % = 100% × (T3-T1)/T1.

**Table 3:** Adjusted mean of BMD at various sites by subgroups of fruit and vegetables intake tertiles ^a^.

BMD	T1 (n = 1029)		T2 (n = 1031)		T3 (n = 1029)		Difference ^b^		ANCOVA	
g/cm^2^	Mean	SE	Mean	SE	Mean	SE	Abs.	%	*P* difference	*P* trend
**Fruit**										
**Citrus fruit** ^a^										
Whole body	1.096	0.003	1.098	0.003	1.106	0.003	0.010	0.91	0.058	**0.025**
Spine (L1–L4)	0.883	0.004	0.884	0.004	0.889	0.004	0.005	0.57	0.641	0.383
Total hip	0.829	0.003	0.831	0.003	0.835	0.003	0.006	0.72	0.397	0.187
Femoral neck	0.684	0.003	0.690	0.003	0.695	0.003*	0.011	1.61	0.028	**0.008**
**Apples, pears, peaches, pineapples, plums**										
Whole body	1.093	0.003	1.100	0.003	1.106	0.003**	0.014	1.28	0.006	**0.001**
Spine (L1–L4)	0.877	0.004	0.889	0.004	0.891	0.004	0.014	1.60	0.043	**0.019**
Total hip	0.823	0.003	0.835	0.003*	0.837	0.003**	0.014	1.70	0.003	**0.002**
Femoral neck	0.681	0.003	0.693	0.003*	0.694	0.003**	0.013	1.91	0.003	**0.003**
**Persimmons, mangoes, durian and melon fruit**										
Whole body	1.098	0.003	1.100	0.003	1.101	0.003	0.003	0.27	0.844	0.560
Spine (L1–L4)	0.884	0.004	0.887	0.004	0.885	0.004	<0.001	<0.01	0.907	0.956
Total hip	0.830	0.003	0.833	0.003	0.832	0.003	0.002	0.24	0.794	0.687
Femoral neck	0.689	0.003	0.691	0.003	0.689	0.003	<0.001	<0.01	0.771	0.993
**Grapes, litchi, and longan**										
Whole body	1.094	0.003	1.104	0.003*	1.101	0.003	0.007	0.64	0.040	0.088
Spine (L1–L4)	0.883	0.004	0.890	0.004	0.883	0.004	0.001	0.11	0.393	0.894
Total hip	0.825	0.003	0.835	0.003	0.834	0.003	0.009	1.09	0.050	**0.047**
Femoral neck	0.684	0.003	0.692	0.003	0.693	0.003	0.008	1.17	0.087	**0.043**
**Bananas**										
Whole body	1.097	0.003	1.099	0.003	1.103	0.003	0.005	0.46	0.457	0.216
Spine (L1–L4)	0.882	0.004	0.885	0.004	0.889	0.004	0.007	0.79	0.488	0.231
Total hip	0.828	0.003	0.832	0.003	0.835	0.003	0.008	0.97	0.235	0.090
Femoral neck	0.685	0.003	0.689	0.003	0.694	0.003	0.009	1.31	0.099	**0.032**
**Other fruits**										
Whole body	1.094	0.003	1.102	0.003	1.104	0.003	0.010	0.91	0.049	**0.019**
Spine (L1–L4)	0.875	0.004	0.893	0.004**	0.888	0.004	0.013	1.49	0.006	**0.030**
Total hip	0.825	0.003	0.833	0.003	0.837	0.003*	0.012	1.45	0.027	**0.008**
Femoral neck	0.683	0.003	0.690	0.003	0.696	0.003**	0.013	1.90	0.008	**0.002**
**Vegetables**										
** Dark green vegetables**										
Whole body	1.098	0.003	1.103	0.003	1.099	0.003	0.001	0.09	0.459	0.776
Spine (L1–L4)	0.884	0.004	0.886	0.004	0.886	0.004	0.002	0.23	0.955	0.803
Total hip	0.830	0.003	0.833	0.003	0.832	0.003	0.002	0.24	0.761	0.614
Femoral neck	0.688	0.003	0.693	0.003	0.687	0.003	-0.001	-0.15	0.255	0.873
** Carrots, peppers, and tomatoes**										
Whole body	1.099	0.003	1.105	0.003	1.095	0.003	-0.004	-0.36	0.064	0.419
Spine (L1–L4)	0.880	0.004	0.896	0.004	0.880	0.004^#^	<0.001	<0.01	0.010	0.947
Total hip	0.828	0.003	0.835	0.003	0.831	0.003	0.003	0.36	0.284	0.563
Femoral neck	0.685	0.003	0.693	0.003	0.690	0.003	0.005	0.73	0.128	0.233
** Light green vegetables**										
Whole body	1.102	0.003	1.098	0.003	1.099	0.003	-0.002	-0.18	0.747	0.600
Spine (L1–L4)	0.885	0.004	0.883	0.004	0.887	0.004	0.002	0.23	0.808	0.736
Total hip	0.831	0.003	0.829	0.003	0.834	0.003	0.002	0.24	0.635	0.625
Femoral neck	0.691	0.003	0.685	0.003	0.693	0.003	0.002	0.29	0.191	0.671
** Melons, eggplant and radish**										
Whole body	1.103	0.003	1.099	0.003	1.097	0.003	-0.006	-0.54	0.402	0.190
Spine (L1–L4)	0.889	0.004	0.882	0.004	0.885	0.004	-0.004	-0.45	0.517	0.459
Total hip	0.833	0.003	0.832	0.003	0.830	0.003	-0.003	-0.36	0.844	0.562
Femoral neck	0.691	0.003	0.689	0.003	0.689	0.003	-0.002	-0.29	0.852	0.584

BMD, bone mineral density. SE, standard error. ANCOVA, analyses of covariance.

* *P*<0.05

** *P*<0.01

***P<0.001, compared with tertile 1

^#^ *P*<0.05, compared with tertile 2.

^,*a*,*b*^: see Table 2

### Odds ratios of the occurrence of osteoporosis according to FV tertiles

Univariate logistic regression analysis showed a significant dose-dependent association between FV intake and the occurrence of osteoporosis (Table 4), although the associations were slightly attenuated after further adjustment of the covariates. The odds ratios (95% confidence interval, CI) of osteoporosis for the highest (vs. bottom) tertile of total FV intake were 0.73 (0.58 to 0.92), 0.37 (0.22 to 0.60), and 0.71 (0.52 to 0.97) at the LS, TH, and FN, respectively, in the multivariate model (*P* trend: <0.001 to 0.031).

**Table 4:** Odds ratios (95% confidence interval) of osteoporosis for the tertiles of fruit and vegetables intake.

	N (%) osteoporosis			Crude odds ratio (95% confidence interval)				Adjusted odds ratio (95% confidence interval)^a^			
	T1	T2	T3	T1	T2	T3	*P* trend	T1	T2	T3	*P* trend
**Total fruit and vegetable intake**											
Spine (L1–L4)	294(28.6)	231(22.4)	222(21.6)	1.00	0.72(0.59–0.88)**	0.69(0.56–0.84)***	**<0.001**	1.00	0.71(0.57–0.89)**	0.73(0.58–0.92)**	**0.008**
Total hip	74(7.2)	44(4.3)	28(2.7)	1.00	0.58(0.39–0.84)**	0.36 (0.23–0.56)***	**<0.001**	1.00	0.54(0.35–0.82)**	0.37(0.22–0.60)***	**<0.001**
Femoral neck	144(14.0)	113(11.0)	94(9.1)	1.00	0.76(0.58–0.98)*	0.62(0.47–0.81)***	**<0.001**	1.00	0.78(0.58–1.04)	0.71(0.52–0.97)*	**0.031**
**Fruit intake**											
Spine (L1–L4)	274(26.6)	245(23.8)	228(22.2)	1.00	0.86(0.70–1.05)	0.78(0.64–0.96)*	**0.018**	1.00	0.87(0.70–1.08)	0.82(0.66–1.03)	0.092
Total hip	70(6.8)	43(4.2)	33(3.2)	1.00	0.60(0.40–0.88)**	0.45(0.30–0.69)***	**<0.001**	1.00	0.63(0.41–0.97)*	0.48(0.30–0.77)**	**0.002**
Femoral neck	134(13.0)	112(10.9)	105(10.2)	1.00	0.81(0.62–1.06)	0.76 (0.58–0.99)*	**0.044**	1.00	0.82(0.61–1.10)	0.89(0.61–1.12)	0.214
**Vegetable intake**											
Spine (L1–L4)	271(26.3)	251(24.3)	225(21.9)	1.00	0.90(0.74–1.10)	0.78(0.64–0.96)*	**0.018**	1.00	0.89(0.71–1.11)	0.80(0.64–1.01)	0.061
Total hip	60(5.8)	51(4.9)	35(3.4)	1.00	0.84(0.57–1.23)	0.57(0.37–0.87)**	**0.010**	1.00	0.84(0.55–1.28)	0.57(0.35–0.92)*	**0.023**
Femoral neck	130(12.6)	125(12.1)	96(9.3)	1.00	0.95(0.73–1.24)	0.71(0.54–0.94)*	**0.018**	1.00	0.97(0.72–1.30)	0.76(0.55–1.05)	0.101

Osteoporosis is defined as a BMD ≤ “mean -2.5 SD” of the reference group. Mean (SD) BMD (g/cm^2^) of reference groups at lumbar spine (L1–L4), total hip, and femur neck are 1.047 (0.110), 0.942 (0.122), and 0.858 (0.120), respectively. Total number of participants are 1029, 1031, and 1029 in tertiles 1–3.

^*a*^ Covariates adjusted for in the multivariate model: age, sex, BMI, educational level, marital status, household income, years since menopause (set at 0 for men), estrogen use (set as no for men), osteoporosis treatment use, physical activities (MET), smoking, passive smoking, tea and alcohol drinking, use of calcium supplement, use of multivitamin supplement, dietary energy, energy-adjusted diet protein and diet Calcium (remove the calcium from FV group being analyzed).; method for covariates = enter.

* *P*<0.05

** *P*<0.01

***P<0.001, Compared with tertile 1.

### The stratified analyses result

In the stratified analyses, the favorable association between FV intake and the occurrence of osteoporosis remained significant in subjects with BMI < 24.0 kg/m^2^ (*P* < 0.05) but not in those who were overweight or obese (BMI≥ 24.0 kg/m^2^; *P* > 0.5) (S4 Table). No significant difference in the associations between other strata stratified by sex, age, education, income, MET, smoking, tea or alcohol consumption, use of calcium or multivitamin supplements, and osteoporosis treatment, was observed (*P*-interaction range: 0.153–0.997) (data not shown).

## Discussions

In this cross-sectional study of 3089 middle-aged and elderly Chinese subjects, we found that greater fruit intake and FV intake had dose-dependent associations with greater BMD and a lower risk of osteoporosis. However, we found no significant association between vegetable intake and BMD. A previous study reported that a decrease of 1 SD in the TH BMD was associated with an 85% increase in the total osteoporotic fracture risk (95% CI, 71% to 101%) [29]. According to this estimate, an increase of 0.0.097 SD in the BMD at the TH when the FV intake was increased from 349 to 685 g/d would result in an 8.3% decrease in the osteoporotic fracture risk in our population. These results suggest that greater FV intake may be beneficial for the prevention of osteoporosis.

Several studies have focused on the role of FV in bone health. Most of these studies were observational and yielded weak favorable associations. Tuck *et al*. first reported the positive association of FV intake with BMD in the elderly in 1999 [30]. Favorable associations between FV intake and BMD were also observed for adult men and women in other observational studies [5–10]. A previous study of 670 postmenopausal women from Hong Kong reported that a daily increase of 100 g in FV intake was associated with increases of 0.0062 g/cm^2^, 0.0098 g/cm^2^, and 0.0060 g/cm^2^ in BMD at the WB, LS, and TH, respectively [8]. The latest finding from a case-control study showed that greater FV intake had an inverse association with the risk of hip fracture (odds ratio for extreme quartiles, 0.25; 95% CI, 0.15 to 0.41) in elderly Chinese subjects [7]. Similar associations were found in the European Prospective Investigation into Cancer and nutrition study (EPIC) [9], the Cohort of Swedish Men study (COSM), and the Swedish Mammography Cohort study (SMC) [5]. Our findings are consistent with those results and support the hypothesis that greater FV intake might be beneficial to bone health.

However, null associations were also found in many other studies or subgroups, especially in cohort studies [30–32] and randomized controlled trials [12–14]. A cohort study (EPIC-Norfolk; 470 men and 474 women; mean follow-up time, 3 years) found no effect of FV, combined or separately, on the rate of BMD loss [32]. In the EPIC-Norfolk study, the bone loss rates were much lower than those found in other studies. The limited study size and large random error in the assessment of BMD changes or fracture incidence might also partially explain the inconsistent results. In addition, regression dilution is common in cohort studies when the exposure variable is measured only at baseline. To the best of our knowledge, only four randomized controlled trials (with less than 60 subjects in each group, intervention time: 12 weeks to 2 years) on FV intake and bone health (bone turnover markers) have been conducted, and three of them yielded a null effect [12–14]. The participants in two of these studies [13, 14] already had a higher FV intake at baseline (more than 3.5 servings of FV and more than 2 servings of fruit) than the general population, which makes detection of the effects of supplemental FV more difficult possibly due to the potential ceiling effect. Some large studies showed that the dose-response protective effects of FV on bone health were observed at the intakes below 3–5 servings/day, but no further benefits were found after 5 servings/day [5, 10]. Therefore, further large and long-term experimental studies in populations with habitual low FV intake are needed to address this issue.

The mechanisms by which bone health is improved by FV have not been thoroughly investigated. The acid-base hypothesis postulates that the acid load is in part buffered by bone tissue, leading to bone absorption and reduced bone density [33]. FV, as a good source of alkaline precursors (e.g., K, Ca, Mg), could neutralize the calciuric effects of acids derived from the diet, as demonstrated in a recent meta-analysis [3]. In addition, vitamin C, vitamin K, and phytochemicals highly enriched in FV are involved in the synthesis of bone matrix. Vitamin C might affect bone mass in the hydroxylation of lysine and proline, which are needed for the formation of stable collagen triple helixes [2]. Vitamin K may play a protective role against age-related bone loss via vitamin K–dependent γ-carboxylation of osteocalcin [2]. Antioxidative nutrients and phytochemicals, such as vitamin C, β-carotene, and other carotenoids found in FV, may improve bone health by scavenging oxygen radicals [34]. In addition, FV is the main source of dietary calcium.

Vegetables showed a weaker association with BMD or osteoporosis than fruit in this study and in previous studies [6, 35], possibly because that vegetables are often consumed in cooked form in Chinese populations and the high intake of sodium with vegetables might accelerate calcium excretion [36] and offset the possible benefit of vegetables. Besides, vegetables were typically cooked by stir-frying, boiling, or steaming in China. This could lead to the substantial loss of water-soluble or heat-sensitive nutrients and phytochemicals and attenuate the association between vegetables and bone health [35]. Furthermore, recent cohort studies showed that in comparison to an intake of 1–3 servings/d (or 80 g/d-240 g/d) of vegetables, less than 1 servings/d (or 80 g/d) or zero intake of vegetables was associated with higher risk of hip fracture but higher intakes did not confer additional benefits [5, 10]. Our study subjects had a relatively high vegetable intake (>300 g/d on average) and <0.3% (8 subjects) of them consumed vegetables <80 g/d. It could be difficult to show a significant association between vegetable and bone health in this population.

In stratified analyses, we found that the association between osteoporosis and total FV intake did not vary by MET or several sociodemographic or behavioral factors, although the limited sample size in some strata makes the detection of correlations difficult. However, the favorable association was not observed in overweight or obese participants. A similar interaction was observed in the Singapore Chinese Health Study of 63,257 men and women, in which an apparent protective effect of total vegetables/carotenoids was seen for lean men (<20 kg/m^2^) but not for their heavier counterparts [37]. A lower BMI has been associated with increased oxidative damage indexed by 8-hydroxy-2’-deoxyguanosine [38]. Subjects with a lower BMI may have higher oxidative stress in their bones that leads to worse bone health, and the antioxidant effects of FV may counteract this mechanism of osteoporosis (for example, the RANKL pathway) related to a low BMI [37]. In addition, heavier individuals had better bone mass, which might leave limited room for FV to show a benefit.

To the best of our knowledge, this is the first large study to explore the association of the intake of FV and subgroups with BMD and osteoporosis in a middle-aged and elderly population. The large study size and higher intake of FV provide greater power to detect a potential association. However, some limitations should be acknowledged. First, the cross-sectional design of the study did not allow inferring causality, although we used average values of dietary intake to better estimate the habitual consumption over the period before the BMD assessments among the follow-up subjects, attenuating the possibility of casual inversion in the majority of study participants. Second, the participants were not a nationally representative sample. The FV intake of 462 g/d in our participants was higher than the 427 g/d cited in the general population in Guangzhou in the 2002 Chinese National Nutritional Survey [39]. Subjects who have high FV intake tend to have healthier lifestyles. We could not fully avoid the possibility that the favorable FV-bone association might be caused by residual confounding although we carefully adjusted for a variety of potential confounders in this study. Furthermore, although the FFQ has been validated, misreporting (under- or over-reporting) of food intake is a common phenomenon in diet assessments [40], which could cause inaccuracy and imprecision in the nutrient and food intake assessed. For this reason, subjects with unreasonably high or low dietary energy intake were excluded from our analyses, and energy adjustment may serve to mitigate some of this error.

## Conclusions

In conclusion, this study showed that greater FV intake has an independent association with a significantly higher BMD and a lower presence of osteoporosis in middle-aged and elderly Chinese subjects with a BMI less than 24.0 kg/m^2^. The favorable association of FV intake with BMD appears mainly attributable to fruit intake. Our findings add to the existing evidence that FV may play a beneficial role in bone health. Long-term FV intervention trials or cohort studies are needed to confirm these findings.

## Supporting Information

S1 Fig Flow chart of the study participants. (TIF)

S1 Table Dietary intake of energy-adjusted fruit and vegetables and the subgroups in each tertile. (DOCX)

S2 Table Mean BMD by tertiles of fruit and vegetables intake. (DOCX)

S3 Table BMI-excluded covariate-adjusted mean of BMD at various sites by fruit and vegetables intake tertiles of the study participant. (DOCX)

S4 Table Odds ratios (95% CIs) of osteoporosis for tertiles of total fruit and vegetables stratified by BMI. (DOCX)
