# Supplementary figures and images for: Greater Intake of Fruit and Vegetables Is Associated with Greater Bone Mineral Density and Lower Osteoporosis Risk in Middle-Aged and Elderly Adults
Source: PLoS One. 2017 Jan 3;12(1):e0168906. doi: 10.1371/journal.pone.0168906 (PMC5207626; doi:10.1371/journal.pone.0168906)

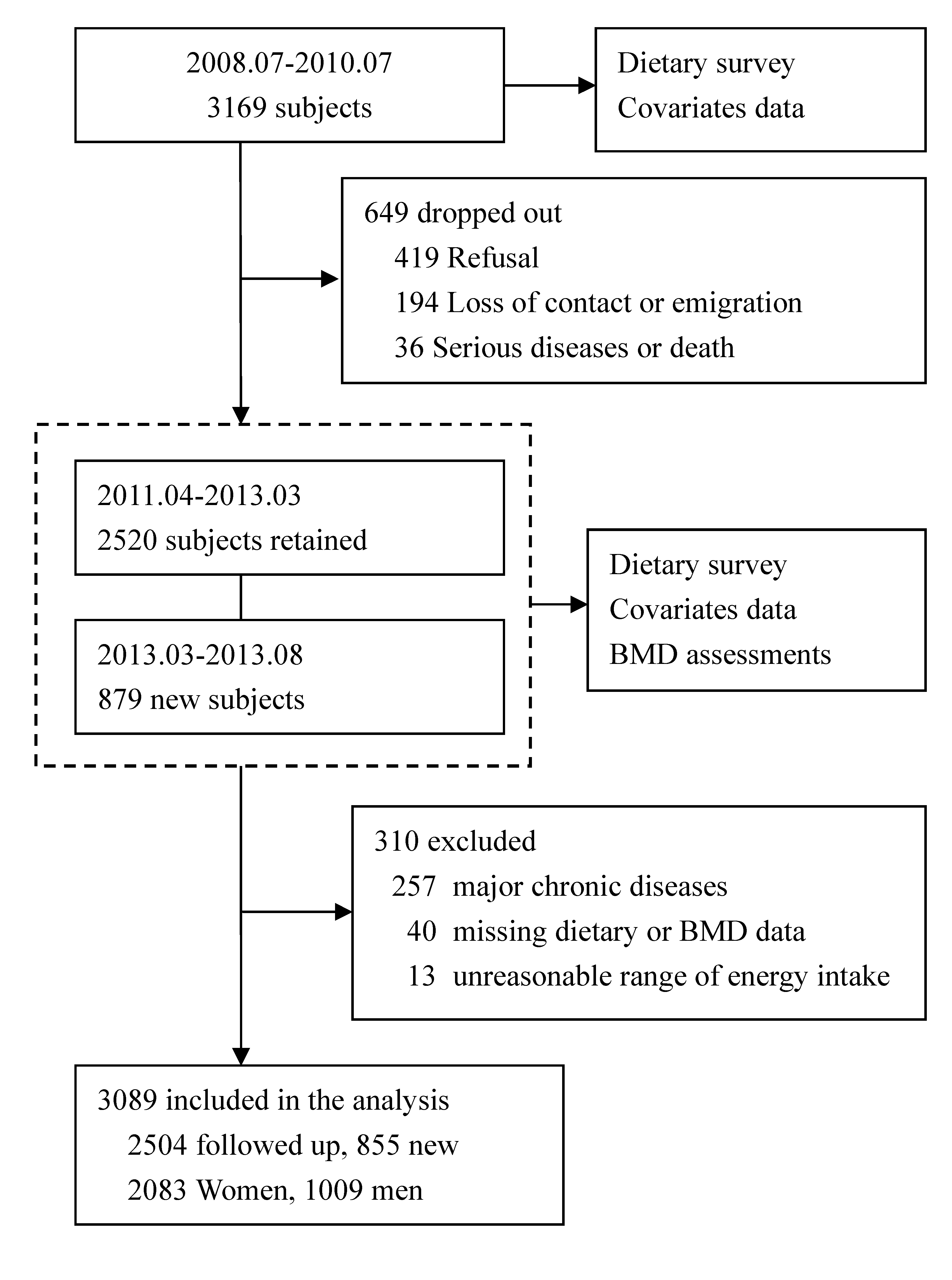

Supplement: S1 Fig — (TIF) [file pone.0168906.s001.tif]
