# Supplementary material for: Greater Intake of Fruit and Vegetables Is Associated with Greater Bone Mineral Density and Lower Osteoporosis Risk in Middle-Aged and Elderly Adults
Source: PLoS One. 2017 Jan 3;12(1):e0168906. doi: 10.1371/journal.pone.0168906 (PMC5207626; doi:10.1371/journal.pone.0168906)
Supplement: S1 Table — (DOCX) [file pone.0168906.s002.docx]

**S1Table.** Dietary intake of energy-adjusted fruit and vegetables and the subgroups in each tertile.

|  | Tertile of intake (g/d) | | | | |
| --- | --- | --- | --- | --- | --- |
|  | T1 |  | T2 |  | T3 |
| **Total fruit and vegetables** | 349 (96.3) |  | 496(67.9) |  | 685 (164) |
| *Total fruit* | 66.9(35.3) |  | 130 (35.2) |  | 222(78.3) |
| Citrus fruit ^a^ | 5.24(5.56) |  | 16.7(7.37) |  | 41.3(27.0) |
| Apples, pears, peaches, pineapples, and plums | 16.9(15.7) |  | 48.4(19.2) |  | 102 (44.5) |
| Bananas | 1.86(3.67) |  | 10.4(8.11) |  | 30.1(23.6) |
| Grapes, lychee, longan | 1.74(1.81) |  | 5.99(3.01) |  | 18.5(17.2) |
| Persimmons, mangoes, durian and melon fruit ^b^ | 2.54(2.65) |  | 8.47(4.00) |  | 23.6(17.1) |
| Other fruit | 0.563(1.39) |  | 5.09(3.99) |  | 21.0(20.4) |
| *Total vegetables* | 242 (75.9) |  | 354 (59.4) |  | 505 (130) |
| Dark-green vegetables ^c^ | 93.6(36.7) |  | 154.5(30.7) |  | 237(67.3) |
| Light-green vegetables ^d^ | 12.4(10.1) |  | 31.0(10.4) |  | 59.9(30.1) |
| Melons ^e^, eggplant and radish | 22.4(13.9) |  | 49.5(17.0) |  | 93.8(43.1) |
| Carrots, tomatoes, peppers | (12.8) |  | 46.5(13.0) |  | 84.5(37.6) |
| Fresh corn | 4.21(3.92) |  | 12.0(4.97) |  | 27.0(16.1) |
| Mushrooms and fungi | 4.34(3.71) |  | 11.8(4.95) |  | 26.8(14.7) |

Data were presented as medians and interquartile range (IQR).

^a^ For example, orange, grapefruit, and lemon

^b^ For example, papaya, cantaloupes, watermelons and other muskmelons

^c^ For example, pak choi, choi sum, lettuce, spinach, Chinese spinach, water spinach, Chinese Kale, mustard, fresh beans.

^d^ For example, broccoli, cabbage, cauliflower, celery, onion and garlic.

^e^ For example, Chinese waxgourd, pumpkin, cucumber, towel gourd, bitter gourd, luffa-smooth loofah, eggplant.
