# Supplementary material for: Greater Intake of Fruit and Vegetables Is Associated with Greater Bone Mineral Density and Lower Osteoporosis Risk in Middle-Aged and Elderly Adults
Source: PLoS One. 2017 Jan 3;12(1):e0168906. doi: 10.1371/journal.pone.0168906 (PMC5207626; doi:10.1371/journal.pone.0168906)
Supplement: S2 Table — (DOCX) [file pone.0168906.s003.docx]

**S2 Table.** Mean BMD by tertiles of fruit and vegetables intake.

| BMD | T1（n=1029） | |  | T2（n=1031） | |  | T3（n=1029） | |  | Difference | |  | ANOVA | |
| --- | --- | --- | --- | --- | --- | --- | --- | --- | --- | --- | --- | --- | --- | --- |
| g/cm^2^ | Mean | SD |  | Mean | SD |  | Mean | SD |  | Abs. | % |  | *P* difference | *P* trend |
| *Total fruit and vegetable intake* | | |  |  |  |  |  |  |  |  |  |  |  |  |
| Whole body | 1.091 | 0.120 |  | 1.101 | 0.113 |  | 1.107 | 0.114^**^ |  | 0.016 | 1.47 |  | **0.008** | **0.002** |
| Spine (L1–L4) | 0.874 | 0.158 |  | 0.888 | 0.153 |  | 0.894 | 0.151^**^ |  | 0.020 | 2.29 |  | **0.011** | **0.003** |
| Total hip | 0.820 | 0.126 |  | 0.833 | 0.121 |  | 0.841 | 0.117^***^ |  | 0.021 | 2.56 |  | **<0.001** | **<0.001** |
| Femoral neck | 0.681 | 0.114 |  | 0.689 | 0.113 |  | 0.690 | 0.112^**^ |  | 0.017 | 2.50 |  | **0.002** | **<0.001** |
| *Fruit intake* |  |  |  |  |  |  |  |  |  |  |  |  |  |  |
| Whole body | 1.093 | 0.117 |  | 1.097 | 0.116 |  | 1.109 | 0.115^**^ |  | 0.016 | 1.46 |  | **0.004** | **0.002** |
| Spine (L1–L4) | 0.875 | 0.157 |  | 0.887 | 0.155 |  | 0.894 | 0.151^*^ |  | 0.018 | 2.06 |  | **0.025** | **0.007** |
| Total hip | 0.823 | 0.124 |  | 0.829 | 0.120 |  | 0.843 | 0.120^*^^*,#^ |  | 0.020 | 2.43 |  | **0.001** | **<0.001** |
| Femoral neck | 0.681 | 0.112 |  | 0.688 | 0.111 |  | 0.700 | 0.112^***^ |  | 0.019 | 2.79 |  | **0.001** | **<0.001** |
| *Vegetable intake* | |  |  |  |  |  |  |  |  |  |  |  |  |  |
| Whole body | 1.099 | 0.118 |  | 1.097 | 0.115 |  | 1.103 | 0.116 |  | 0.005 | 0.45 |  | 0.445 | 0.363 |
| Spine (L1–L4) | 0.881 | 0.154 |  | 0.883 | 0.156 |  | 0.892 | 0.152 |  | 0.011 | 1.25 |  | 0.197 | 0.095 |
| Total hip | 0.827 | 0.125 |  | 0.828 | 0.119 |  | 0.840 | 0.120^*^ |  | 0.013 | 1.57 |  | **0.029** | **0.016** |
| Femoral neck | 0.686 | 0.114 |  | 0.687 | 0.111 |  | 0.696 | 0.110 |  | 0.010 | 1.46 |  | 0.098 | **0.048** |

BMD, bone mineral density; ANOVA, analysis of variance.

Compared with tertile 1: * *P*<0.05; ** *P*<0.01; ****P*<0.001

Compared with tertile 2:^#^ *P*<0.05
