# Supplementary material for: Greater Intake of Fruit and Vegetables Is Associated with Greater Bone Mineral Density and Lower Osteoporosis Risk in Middle-Aged and Elderly Adults
Source: PLoS One. 2017 Jan 3;12(1):e0168906. doi: 10.1371/journal.pone.0168906 (PMC5207626; doi:10.1371/journal.pone.0168906)
Supplement: S3 Table — (DOCX) [file pone.0168906.s004.docx]

**S3 Table** . BMI-excluded covariate-adjusted mean of BMD at various sites by fruit and vegetables intake tertiles of the study participant. *^a^*

| BMD | T1(n=1029) | | T2(n=1031) | | | | T3(n=1029) | | Difference *^b^* | | |  | ANCOVA | |
| --- | --- | --- | --- | --- | --- | --- | --- | --- | --- | --- | --- | --- | --- | --- |
| g/cm^2^ | Mean | SE |  | Mean | SE |  | Mean | SE |  | Abs. | % |  | *P* difference | *P* trend |
| *Total fruit and vegetable intake* | | |  |  |  |  |  |  |  |  |  |  |  |  |
| Whole body | 1.093 | 0.003 |  | 1.102 | 0.003 |  | 1.104 | 0.003^*^ |  | 0.012 | 1.10 |  | **0.025** | **0.010** |
| Spine (L1–L4) | 0.875 | 0.005 |  | 0.888 | 0.004 |  | 0.893 | 0.004^*^ |  | 0.018 | 2.06 |  | **0.020** | **0.007** |
| Total hip | 0.821 | 0.003 |  | 0.833 | 0.003^*^ |  | 0.840 | 0.003^***^ |  | 0.019 | 2.31 |  | **0.001** | **<0.001** |
| Femoral neck | 0.681 | 0.003 |  | 0.690 | 0.003 |  | 0.697 | 0.003^**^ |  | 0.016 | 2.35 |  | **0.003** | **0.001** |
| *Fruit intake* |  |  |  |  |  |  |  |  |  |  |  |  |  |  |
| Whole body | 1.095 | 0.003 |  | 1.097 | 0.003 |  | 1.108 | 0.003^**,#^ |  | 0.013 | 1.19 |  | **0.004** | **0.002** |
| Spine (L1–L4) | 0.878 | 0.004 |  | 0.886 | 0.004 |  | 0.892 | 0.004 |  | 0.014 | 1.59 |  | 0.097 | **0.031** |
| Total hip | 0.825 | 0.003 |  | 0.828 | 0.003 |  | 0.842 | 0.003^**,#^ |  | 0.017 | 2.06 |  | **0.001** | **<0.001** |
| Femoral neck | 0.682 | 0.003 |  | 0.688 | 0.003 |  | 0.699 | 0.003^***,#^ |  | 0.017 | 2.49 |  | **0.001** | **<0.001** |
| *Vegetable intake* | |  |  |  |  |  |  |  |  |  |  |  |  |  |
| Whole body | 1.100 | 0.003 |  | 1.098 | 0.003 |  | 1.101 | 0.003 |  | 0.002 | 0.18 |  | 0.689 | 0.729 |
| Spine (L1–L4) | 0.881 | 0.005 |  | 0.884 | 0.004 |  | 0.891 | 0.005 |  | 0.010 | 1.14 |  | 0.252 | 0.113 |
| Total hip | 0.827 | 0.003 |  | 0.828 | 0.003 |  | 0.839 | 0.003^*^ |  | 0.012 | 1.45 |  | **0.024** | **0.013** |
| Femoral neck | 0.685 | 0.003 |  | 0.688 | 0.003 |  | 0.695 | 0.003 |  | 0.010 | 1.46 |  | 0.076 | **0.031** |

BMD, bone mineral density. SE, standard error. ANCOVA, analyses of covariance.

Compared with tertile 1: * *P*<0.05; ** *P*<0.01; ****P*<0.001

Compared with tertile 2:^#^ *P*<0.05

*^a^* Covariates adjusted for in the multivariate model: age, sex, educational level, marital status, household income, years since menopause (set at 0 for men), estrogen use (set as no for men), osteoporosis treatment use, physical activities (MET), smoking, passive smoking, tea and alcohol drinking, use of Calcium supplement, use of multivitamin supplement, dietary energy, energy-adjusted diet protein and diet Calcium (remove the calcium from FV group being analyzed). BMI was excluded from the covariates.

*^b^* Difference between tertile 3 and tertile 1: Abs., absolute mean difference (T3 – T1); %, relative difference, % = 100 % × (T3 - T1)/T1.
