# Supplementary material for: Greater Intake of Fruit and Vegetables Is Associated with Greater Bone Mineral Density and Lower Osteoporosis Risk in Middle-Aged and Elderly Adults
Source: PLoS One. 2017 Jan 3;12(1):e0168906. doi: 10.1371/journal.pone.0168906 (PMC5207626; doi:10.1371/journal.pone.0168906)
Supplement: S4 Table — (DOCX) [file pone.0168906.s005.docx]

**S4 Table .** Odds ratios (95% CIs) of osteoporosis for tertiles of total fruit and vegetables stratified by BMI.

|  | N (%) osteoporosis | | |  | Adjusted OR (95%CI)^a^ | | | | | | | | *P* interaction |
| --- | --- | --- | --- | --- | --- | --- | --- | --- | --- | --- | --- | --- | --- |
|  | T1 | T2 | T3 |  | T1 | | T2 | | T3 | | *P* trend | |  |
| *BMI < 24.0 kg/m^2^* | | | | | | | | | | | | | |
| Spine (L1–L4) | 234(37.1) | 165(28.1) | 157(26.9) | 1.00 | | 0.60(0.45-0.78)*** | | 0.63(0.48-0.84) ** | | **0.001** | | | 0.111 |
| Total hip | 67(10.6) | 32(5.4) | 20(3.4) | 1.00 | | 0.39(0.24-0.64)*** | | 0.29(0.16-0.51)*** | | **<0.001** | | | **0.016** |
| Femoral neck | 121(19.2) | 82(13.9) | 74(12.7) | 1.00 | | 0.64(0.45-0.91) * | | 0.66(0.46-0.95) * | | **0.020** | | | 0.935 |
| *BMI* ≥ *24.0 kg/m^2^* | | | | | | | | | | | | | |
| Spine (L1–L4) | 60(15.1) | 66(14.9) | 65(14.6) | 1.00 | | 1.01(0.67-1.50) | | 1.02(0.68-1.55) | | 0.992 | |  | |
| Total hip | 7(1.8) | 12(2.7) | 8(1.8) | 1.00 | | 1.37(0.48-3.87) | | 0.81(0.26-2.58) | | 0.818 | |  | |
| Femoral neck | 23(5.8) | 31(7.0) | 20(4.5) | 1.00 | | 1.34(0.72-2.48) | | 0.96(0.48-1.92) | | 0.887 | |  | |

OR, odds ratio. CI, confidence interval.

Osteoporosis is defined as a BMD 2.5 SD or more below the mean of reference group. Mean (SD) BMD (g/cm2) of reference groups at lumbar spine (L1–L4), total hip, and femur neck are 1.047 (0.110), 0.942 (0.122), and 0.858 (0.120), respectively. Total number of participants in tertile 1, 2 and 3 are 1029, 1031, and 1029, respectively.

^a^ Covariates adjusted for in the multivariate model: age, sex, educational level, marital status, household income, years since menopause (set at 0 for men), estrogen use (set as no for men), osteoporosis treatment use, physical activities (MET), smoking, passive smoking, tea and alcohol drinking, use of calcium supplement, use of multivitamin supplement, dietary energy, energy-adjusted diet protein and diet Calcium (remove the calcium from FV group being analyzed).; method for covariates = enter

Compared with tertile 1: * *P*<0.05; ** *P*<0.01; ****P*<0.001
